# Larvicidal activity of *Agave sisalana *against *Aedes aegypti *mosquito, the dengue vector

**DOI:** 10.1186/1753-6561-8-S4-P5

**Published:** 2014-10-01

**Authors:** Fabiola Nunes, Louise Guimarães, Debora Lacerda, Sandra Mascarenhas, Valdir Braga

**Affiliations:** 1 Federal University of Paraiba, João Pessoa, Paraíba, Brazil

## Background

Dengue is a viral systemic disease caused by an arboviral of Flaviviridae family, affecting about a 700 thousand cases per year in Brazil. It is endemic in tropical regions such as Southeast Asia, South Pacific, East Africa, Caribbean and Latin America. The disease is transmitted by *Aedes aegypti *(Linnaeus, 1762), a mosquito that is the main target for the disease control through strategies ranging from the larval to the adult combat. The larvicides commonly used to combat the vector, besides being toxic, present drop in larvicide efficacy since the *A. aegypti *larvae has developed resistance to these products. Thus, the search for new active principles that are effective in combating the mosquito is required. In this sense, *Agave sisalana *is a plant that is produced in several states in the Brazilian northeast region, which is used in the sisal industry. Only 5% of the plant is recovered, and its residual liquid completely wasted. In this way, the aim of this research project was to investigate the larvicidal action of the juice of *Agave sisalana *against larvae of *A. aegypti*.

## Methods

In larvicidal activity assays, fourth stage *A. aegypti *larvae were exposed to different concentrations (1, 3, 6, 6.5, 10 and 20 mg/mL) of *A. sisalana *liquid waste dissolved in water, during 24 hours. *The control group consisted of 20 fourth stage larvae, exposed to tap water for 24 hours. The experiment was performed in triplicate*. The histopathological analysis was performed by staining with HE.

## Results and conclusions

The *A. sisalana *concentrations of 1 and 3 mg / mL did not kill any larvae. However, the concentrations of 6, 6.5, 10 and 20 mg / ml respectively killed 78, 78, 88 and 100% of the larvae within 24 hours. After the larvicidal activity assays, it was possible to determine the LC50 that was 5.9 mg/mL. The histological alterations were confirmed by histopathological analysis, which showed lyses of the mesentery epithelial cells of larvae as well as peritrophic membrane destruction. In this way, the *A. sisalana *liquid waste constitutes an effective alternative and economically feasible for the dengue vector combat. The outcomes of our research resulted in the patent.
